# Tailoring and Evaluating an Intervention to Support Self-management After Stroke: Protocol for a Multi-case, Mixed Methods Comparison Study

**DOI:** 10.2196/37672

**Published:** 2022-05-06

**Authors:** Marie Elf, Erika Klockar, Maya Kylén, Lena von Koch, Charlotte Ytterberg, Lars Wallin, Tracy Finch, Catharina Gustavsson, Fiona Jones

**Affiliations:** 1 School of Health and Welfare Dalarna University Falun Sweden; 2 Department of Health Sciences Lund University Lund Sweden; 3 Department of Neurobiology, Care Sciences and Society Karolinska Institutet Stockholm Sweden; 4 Theme Neuro Karolinska University Hospital Stockholm Sweden; 5 Women’s Health and Allied Health Professionals Theme Medical Unit Occupational Therapy and Physiotherapy Karolinska University Hospital Stockholm Sweden; 6 Department of Nursing, Midwifery and Health Faculty of Health and Life Sciences Northumbria University Newcastle upon Tyne United Kingdom; 7 Center for Clinical Research Dalarna Uppsala University Falun Sweden; 8 Department of Public Health and Caring Sciences Uppsala University Uppsala Sweden; 9 Faculty of Health and Social Care Sciences Kingston University and St George's, University of London London United Kingdom

**Keywords:** self-management, self-management support, self-efficacy, stroke rehabilitation, home rehabilitation, person-centered care

## Abstract

**Background:**

Self-management programs are recognized as a valuable approach to supporting people with long-term conditions, such as stroke, in managing their daily lives. Bridges Self-Management (Bridges) focuses on how practitioners interact and support patients’ confidence, skills, and knowledge, and it is an example of a complex intervention. Bridges has been developed and used across multiple health care pathways in the United Kingdom and is theoretically informed by social cognition theory and self-efficacy principles. Evidence shows that self-management programs based on the construct of self-efficacy can be effective. There is still much to learn about how health care services or pathways should implement support for self-management in a sustainable way and whether this implementation process is different depending on the context or culture of the team or service provided.

**Objective:**

The aim of this study is to tailor and evaluate an intervention (Bridges) to support self-management after stroke in a Swedish context.

**Methods:**

We will use a pretest-posttest design with a case study approach to evaluate the feasibility and implementation of self-management support in two stroke settings. This project includes a complex intervention and depends on the actions of individuals, different contexts, and the adaptation of behavior over time. A mixed methods approach was chosen to understand both outcomes and mechanisms of impact. Data collection will comprise outcome measurements and assessment tools as well as qualitative interviews. Data will be collected concurrently and integrated into a mixed methods design.

**Results:**

Recruitment and data collection for the first site of the project ran from September 1, 2021, to January 17, 2022. The intervention at the first site was conducted from November 1, 2021, to March 5, 2022. The evaluation will start after the implementation phase. The second site has been recruited, and the baseline data collection will start in spring 2022. The intervention will start in early autumn 2022. Data collection will be completed by the end of 2022.

**Conclusions:**

This study represents a unique, highly relevant, and innovative opportunity to maximize knowledge and minimize practice gaps in rehabilitation stroke care. The study will produce robust data on the intervention and in-depth data on the contextual factors and mechanisms related to the feasibility of the intervention and for whom it is feasible. Bridges has been used in the United Kingdom for more than 10 years, and this study will explore its contextualization and implementation within a Swedish stroke environment. The evaluation will study results at the patient, staff, and organizational levels and provide recommendations for the adoption and refinement of future efforts to support self-management.

**International Registered Report Identifier (IRRID):**

DERR1-10.2196/37672

## Introduction

### Background

Self-management programs are recognized as a valuable approach to supporting people with long-term conditions, such as stroke, to manage their daily lives [1,2]. The essential skills that support successful self-management are also considered critical when navigating the transition from acute illness with structured care in hospital to discharge home [3,4]. However, there is relatively little knowledge and research about how health care services or pathways should implement support for self-management in a sustainable way and whether this implementation process is different depending on the context or culture of the team or service provided [5]. In this study, as part of the SELMA (Self-Management) project, we will tailor and evaluate an intervention developed in the United Kingdom to support self-management after stroke.

Stroke is an acute condition but can have a long-term impact on individuals and families; despite a reduction in the incidence through advances in prevention and emergency care, support for life after stroke and the long-term effects on the individual and families remain inadequate in most health care systems in Europe [6]. Recent health care policy discourses have accelerated efforts to minimize hospital stays and provide more care in the community and the patient’s home [7,8], which can be challenging. There are high expectations for individuals and family members to manage their rehabilitation and aftercare, including the coordination of services [9]. In addition, the intensity of services in the community can also be limited [10]. This highlights a real need to initiate high-quality self-management support earlier in the stroke pathway to enable individuals and families to manage the transition from hospital to home and live well after the experience of stroke.

Definitions of what constitutes self-management support vary, but it has been conceptualized as a process in which the person living with a long-term condition, such as stroke, is supported to be an active partner in their recovery process [11]; this definition has been supported by the World Health Organization [12]. Self-management has also been described as a fundamental part of person-centered care [13], which aims to give people opportunities to improve and manage their health according to their beliefs, values, and preferences. Person-centered care also supports the premise that people should be given tools and strategies to recognize and develop their strengths and abilities to live independent and satisfying lives as much as is possible [14-16]. In that sense, the concept of person-centered care is strongly related to person-centered self-management support, which prioritizes and encourages patients to define their health outcomes according to what is most meaningful and important [17]. This is supported by research showing that recovery after a stroke can be enhanced when the person and his or her family or care network work collaboratively with health care professionals and are empowered to define their own goals and activities to support recovery [13].

While there is a move toward more collaborative health care and partnership working models, some critics of self-management programs have highlighted their limitations, particularly when they are professionally led and limited to didactic methods of providing health education to individuals and their families [18,19]. Self-management methods have also been criticized for not reaching groups with low health literacy or cognitive or communication impairments [20-22]. In addition, self-management can be conceptualized as a moral responsibility, contingent on personal agency without considering the person’s and family’s circumstances, social capital, networks, health literacy, ethnicity, and culture [22].

Given the high numbers of people who experience cognitive or communication problems and mood disorders poststroke [23,24], there is a need to develop self-management programs that are more inclusive and to find creative ways to support individuals’ knowledge, skills, and confidence to live well with their condition [25]. According to a Cochrane review in 2016, self-management programs based on the construct of self-efficacy are the most effective in changing people’s psychological state and quality of life poststroke [26]. Self-efficacy is a key construct in social cognitive theory [27]. It is defined as people’s beliefs about their capabilities to produce designated levels of performance that influence other events that affect their lives [28].

Self-efficacy beliefs can determine how people feel, think, motivate themselves, and behave concerning their health by determining the goals people set, how much effort they invest in achieving those goals, and their resilience when faced with difficulties or failure. Self-efficacy can be considered a mediator and an outcome, and studies have shown a relationship between self-efficacy, activity performance, disability, mood, and quality of life poststroke [29]. Currently, self-management programs that are theoretically informed by behavior change theories, such as social cognitive theory, show a more significant impact [25,26,30].

Self-management support integrated into everyday health care practice could be one way to avoid the constructions and practice of self-management as an add-on to health care [31-33], but the study of the implementation of programs into service settings, such as a stroke pathway, remains limited. Implementation science is an evolving but established field that provides theories, frameworks, and methodologies for investigating implementation challenges and that contributes to the identification of solutions [34].

The implementation of new ways of working in health care is always context dependent, and careful consideration is required to develop feasible implementation strategies [35]. The intervention in this study is a model of personalized self-management support—Bridges Self-Management (Bridges) [25,31]—and is defined as a complex, as it is characterized by several interacting components that will be implemented in a multidisciplinary team. According to the Medical Research Council (MRC) [35], “An intervention might be considered complex because of properties of the intervention itself, such as the number of components involved; the range of behaviours targeted; expertise and skills required by those delivering and receiving the intervention; the number of groups, settings, or levels targeted; or the permitted level of flexibility of the intervention or its components.” The Bridges intervention is a complex intervention because it targets a range of behaviors that include new working methods and may vary greatly across settings in terms of the professional groups involved and patient populations. The SELMA project will explore the process of the implementation of using Bridges. Process evaluation studies can help address how an intervention works and why it does not work in different contexts. Understanding the process and context can help improve fidelity to the core elements that drive an intervention’s effectiveness and adaptability to the local context.

As a complex intervention, Bridges [25] focuses on how practitioners interact and support confidence, skills, and knowledge, and it is theoretically informed by principles to support self-efficacy. Health care practitioners support individuals in gaining confidence in self-management using specific strategies and coaching language integrated into everyday practice. First established for stroke, Bridges is feasible in community and acute health care settings. In this study, Bridges will be tailored to the Swedish service setting and evaluated using a mixed methods and process evaluation study design.

### Aims and Research Questions

The proposed project aims to evaluate a self-management program intervention and study the implementation process. The specific aims are to explore the following:

1. The feasibility of refining Bridges training for stroke teams and integrating it into Swedish stroke settings.
2. The self-efficacy, health, well-being, self-management, and perceived participation in rehabilitation of patients with stroke pre- and postimplementation.
3. The conceptualization and description of the intervention as a self-management approach by persons with stroke and staff.
4. The ability of the intervention to change the nature of interactions between patients and staff.
5. The critical mechanisms in the implementation process required for integrating self-management support at the individual and ward levels.

## Methods

### Study Design

We will use a pretest-posttest design with a case study approach to evaluate the feasibility and implementation of self-management support in two stroke care settings. Case studies can provide rich data and are particularly useful when understanding the implementation of a complex intervention in a real-world setting in which the process or context cannot be controlled [36]. As this project includes a complex intervention and depends on the actions of individuals, different contexts, and the adaptation of behavior over time [37], we chose a mixed methods approach to understand both the outcomes and mechanisms of impact. The data collection will comprise outcome measurements by self-assessment questionnaires and qualitative interviews. Thus, quantitative and qualitative data will be collected concurrently and integrated into a convergent mixed methods approach [38].

Normalization process theory (NPT) will guide data collection, analysis, and interpretation [39]. NPT focuses on the work done by staff collectively to understand the processes by which a complex health care intervention is or is not implemented, embedded, and integrated into practice [28]. NPT has been applied in health care settings [40], including stroke care and rehabilitation [40-42], allowing us to compare our findings with previous studies. NPT is a sociologically informed theory of how new interventions in health care and social care are implemented and embedded as normal practice. Its primary focus is on how different groups of participants involved in the process of implementation *work together* to achieve a practice that is being implemented through four fundamental mechanisms that affect whether a new practice or way of working becomes normalized and embedded in everyday practice. These mechanisms relate to activity performed with the following objectives:

1. To make sense of the practice change and gain a shared understanding of the purpose and value of the change and how the new practice differs from previous practice (coherence).
2. To participate and have sustained engagement in the activity (cognitive participation).
3. To successfully work together with the new practice within its setting (collective action).
4. To reflect on and appraise the impacts of the activity in ways that can be used to improve the process for those involved (reflexive monitoring).

In developing the study protocol, we followed the SPIRIT (Standard Protocol Items: Recommendations for Interventional Trials) statement [43]. In addition, the description of the intervention follows the Template for Intervention Description and Replication [44]. We also use MRC’s guidelines for process evaluation [37], which identify aspects necessary for the sustainability of implementation in various contexts.

### Study Phases

The project consists of three phases (Multimedia Appendix 1). The aim of the first phase is to plan the implementation by exploring the usual practice. In this prephase, data about the provision of standard stroke care within the organization in addition to patients’ data will be collected to learn about and understand the service within the included organizations; the data will thereby be used to support the implementation of the intervention by the principles of the NPT [39].

To tailor the intervention to the Swedish context, the UK Bridges team will hold additional discovery interviews to explore existing experiences of self-management support, goal setting, and discharge planning methods, as well as any critical issues regarding staff caseloads and organizational challenges. Staff taking part in the training will be asked to provide case studies or personas about patients they are currently working with or have worked with in the past that depict self-management successes and challenges. These case studies will be used to contextualize training in the Swedish setting.

The second phase involves the training of the staff according to the Bridges program, followed by an implementation phase, where the rehabilitation team will implement the intervention (ie, Bridges) in their everyday clinical practice. The implementation phase will last approximately 6 months, which will allow the staff to adopt the intervention and embed it into routine practice. The third phase will involve analysis and reporting on the project’s implementation and its outcomes.

### Logic Model of the Study

The logic model (Multimedia Appendix 2) that guides the evaluation of the intervention was developed based on the MRC Framework for the Development and Evaluation of RCTs (randomized controlled trials) for Complex Interventions to Improve Health [37] and the NPT [39].

### Participating Stroke Units, Staff, and Patients

The study will be conducted at two stroke units in Sweden. We will purposefully recruit stroke units that provide rehabilitation across inpatient and home settings. The 3-year study will be conducted from 2021 to 2023 and will have a mix of multidisciplinary skills.

### Participants

The participants in the study will be staff and patients in stroke settings. In regard to the staff, there will be different numbers of staff who carry out the workshop for the intervention depending on the staff size. We expect most people in a staff group to participate. Those who participate are expected to attend all workshops, participate in the discussions, and carry out the intervention tasks. The staff groups will be mixed based on the staff composition of the unit (ie, nurses, physiotherapists, and occupational therapists).

We will also interview approximately 15 staff members in each of the two units to answer questions regarding the organization and integration of self-management in clinical work before and after the implementation of the intervention. The inclusion criterion for the participants in these interviews will be that they should have been working in rehabilitation for at least 6 months.

A total of 40 patients will be recruited prior to the implementation of the intervention in the standard care context (ie, usual rehabilitation), and 40 will be recruited after the intervention has been implemented in the units (20 at each site). As such, the participants in the preintervention patient group will be different from the participants in the patient group after the implementation of the intervention. The inclusion criteria for the persons with stroke will be as follows: aged 18 years of age or older, 3 to 6 months after stroke onset, discharge from rehabilitation at the hospital or day care, fulfillment of the criteria for a clinical diagnosis of stroke, ability to comprehend study goals and procedures, and knowledge of the Swedish language to such a degree that no interpreter will be required. The participants will also identify a family member who will be asked to participate. The time frame of 3 to 6 months after stroke onset was chosen based on the expectation that patients’ physical recovery would be stabilized. However, they might still face difficulties in everyday life that have become evident after engaging in daily activities in their environment after discharge.

### Recruitment Procedures and Informed Consent

Initially, stroke unit staff from participating stroke units will be asked to identify potential stroke participants based on the inclusion criteria (ie, diagnosis, age, and level of verbal communication). Patients who fulfill the inclusion criteria will receive an information letter; they will be asked if they are interested in participating in the study and if they will allow a person from the research group to contact them. Within 1 to 2 weeks, potential participants will be contacted by telephone, and those who express an interest in participating will be scheduled for an interview. Before the interview, all self-reported instruments will be sent home to the person so they can fill in the questionnaires before the interviews. At the beginning of each interview, the participants will be informed about the study again and will have the opportunity to ask questions. Informed consent will be recorded.

The recruitment of stroke units will be based on suitability (ie, they have home rehabilitation and the teams consists of multidisciplinary professions). The staff at the included sites will receive an information letter about the study, and they will give their written informed consent to participate.

### Description of the Intervention

In this project, the intervention is conceptualized as a staged approach to training staff using Bridges and implementing key principles and strategies within everyday practice with stroke patients. The training will be delivered online and weekly using a staged approach across nine sessions, each lasting between 60 and 90 minutes. Every session will cover critical themes, such as emphasizing the importance of the patient’s narrative, past life experiences, skills, and assets; enhancing self-efficacy through mastery and vicarious experiences; supporting key self-management skills, such as goal setting and reflection; and exploring hopes and fears as motivators and drivers for action. The staff will have key activities to implement in their work after each session. Individually and collectively, they will agree on strategies for implementation in their everyday work and ways to sustain support for self-management posttraining. The training will be co-delivered by an experienced health care academic and practitioner and a person with lived experience of an acute and long-term condition. Staff will receive weekly reminders about key aspects of each session and reminders about their targets for putting Bridges into action. They will also receive access to additional resources, such as an interactive handbook, key evidence, posters, and crib sheets. Staff will be provided with support by an internal, educated, self-management facilitator (ie, champion).

### Outcomes and Data Collection Methods

#### Process Evaluation

Data collection to investigate the implementation, feasibility, role of the context, and influencing mechanisms will take place according to the MRC process evaluation [35]. Data will be collected through individual staff interviews, observations, and researcher-reflective field notes to explore factors that might affect the implementation results. The interview guide for the staff interviews was designed to identify factors that may affect the implementation of Bridges. Questions such as “How do you support patients in self-management?” will be asked. Contextual issues, such as leadership, staff ratio, and organization, will be collected by observations, documents, and interviews with staff and managers.

Researchers will take field notes to observe the sessions throughout the intervention process. The notes will contain observations of how the training sessions and communication work. These data will be complemented by interviews with the Bridges team, who will provide the intervention. The interviews will cover experiences of the intervention, such as delivery method, dose, content, and specific components. The interviews will also cover how closely the participants follow the intervention guide.

The experiences of the intervention from the staff’s perspective will be collected through interviews after the intervention. The questions will cover experiences of the intervention, such as delivery method, dose, and the content of the sessions. In addition, a sample of interactions between patients and staff will be observed using an observational framework for case studies and implementation studies developed by Morgan et al [45]. For example, the researchers will note where the meeting takes place, who participates, what it is about, and how self-management is addressed. The observations will be complemented with interviews to gather the staff’s experiences of their work processes before and after the intervention.

The interviews will be recorded and transcribed verbatim.

#### Experienced Changes

To explore the staff’s experiences of the intervention and any potential changes in working methods after the intervention, interviews will be conducted before and after the intervention. The interviews will be semistructured and will capture the staff’s experiences of their work processes and use of self-management strategies in daily work. Questions will cover the work process and how staff support patients’ self-management in a person-centered way. In addition, questions about how they support patient participation will also be asked.

We will also strive to capture patients’ experiences of how the staff supports self-management and provides opportunities to participate before and after the intervention. Questions will be asked to patients about how they experience the staff supporting them in self-management strategies and the way the staff create opportunities for them to be involved in their care and rehabilitation. The same questions will be asked to each cohort of patients before and after the intervention.

The interviews will be recorded and transcribed verbatim.

#### Outcomes

We will evaluate outcomes at two time points—pre- and postimplementation of the self-management program—using self-reported measurements and interviews pre- and postimplementation. Data from the staff will be collected before and after the intervention in each setting. Data will be collected from patients and relatives in two cohorts before and after the intervention.

#### Staff Data

The following demographic data will be collected from the staff: sex, age, professional occupation, number of years in stroke care and rehabilitation, and the participating site.

Qualitative interviews will be completed to explore the staff’s knowledge, skills, attitudes, and experiences in performing self-management support. The interviews will be semistructured and conducted before and after the intervention. The questions will capture experiences of self-management and the rehabilitation process as well as how the process affects their work. After the intervention, questions will be added about the experience of the intervention. Issues will specifically focus on how the staff adopt strategies for self-management in their contact with patients. The interviews will also explore the experiences of previous uses and potential news and insights into necessary changes after the intervention. The interviews will be recorded and transcribed verbatim.

Integration of self-management in usual practice will be assessed using the Swedish version of the Normalization Measure Development questionnaire (NoMAD) [46], which measures practitioners’ perceptions of the implementation activity level on key NPT-informed domains of work related to the embedding of the intervention: constructs of coherence, cognitive participation, collective action, and reflexive monitoring, as described in the Introduction section. The original version of the NoMAD contains 23 items, and it has been validated across various implementation projects, showing good psychometric properties [46].

#### Patient Data

We will evaluate outcomes at two time points—pre- and postimplementation of the self-management program—by asking two open-ended questions and using self-reported measurements before and after the implementation. The two questions will be as follows: (1) Can you tell me how the staff supported you in gaining confidence, skills, and knowledge [to self-manage]? and (2) Can you tell me how the staff created opportunities for you to be involved in your care and treatment? In addition, standardized self-reported measures that reflect the mechanism of impact, such as self-efficacy for self-management measured by the Stroke Self-efficacy Questionnaire (SSEQ) [47], will be used to measure individual confidence in performing activities after stroke. The SSEQ consists of 13 items that measure two separate elements of self-efficacy. Items 1 to 8 reflect self-efficacy in different activities, and items 9 to 13 reflect self-efficacy in self-management. Each item is scored on a 4-point scale, where 0 means “not at all confident” and 3 means “very confident.” The answer reflects the stroke patient’s confidence in the separate items. The total score ranges from 0 to 39, and this number is then divided by the number of items that have been answered. A higher total score suggests stronger perceived self-efficacy. The scale has been validated and used internationally in self-management studies [31,48].

Perceived health will be measured using a single item from the five-level EQ-5D (EQ-5D-5L) instrument [49], which captures patients’ perceived health at the moment. The EQ-5D-5L includes a visual analog scale that records the respondent’s self-rated health status on a graduated scale from 0 to 100, with higher scores indicating higher health. In addition, experiences of participation will be evaluated by CollaboRATE [50], a 3-item measure of the shared decision-making process, where items include the following: (1) How much effort was made to help you understand your health situation? (2) How much effort was made to listen to the things that matter most to you regarding your health situation? and (3) When you chose what to do next, how much effort was put into considering what is most important to you? The patients will respond on a scale from 0 (“none”) to 9 (“everything”).

Additionally, the Stroke Impact Scale-16 (SIS-16) will be employed to assess self-reported physical function [51]. The SIS-16 is a questionnaire focused on quality-of-life levels related to physical function. Thus, persons are asked to rank the difficulty they experienced during the last 2 weeks when performing 16 skills related to four physical domains (ie, strength, hand function, mobility, and activities of daily living); the difficulty is ranked on a 5-point Likert scale, ranging from 1 (“inability to complete the item”) to 5 (“not difficult at all”). The scores are transformed on a scale from 0 to 100. A higher score indicates better levels of subjective health-related quality of life. The SIS-16 has shown good psychometric validity in stroke studies [52].

We will also use a specially developed scale for self-management, co-designed with stroke survivors and used across the United Kingdom in stroke improvement work and Bridges [53]. The short questionnaire contains eight questions about how patients experience and understand their situation after stroke and how they can use self-care activities. The patient responds on a scale from 1 (“not at all”) to 10 (“entirely”). Example items are as follows: (1) “I understand what caused my stroke,” (2) “I understand why my stroke affected me in the way that it has,” (3) “Right now, I feel confident that I can cope with the ups and downs that can follow a stroke,” (4) “My wishes and priorities were respected when the care staff and I set goals and planned for my care and rehabilitation,” and (5) “I feel confident about what I need to do to continue to improve now that I have been discharged from the hospital.”

Demographic data such as sex, age, diagnosis, and level of stroke burden will also be collected from participating patients. A detailed data collection plan with all the measures is presented in Table 1 [46-53].

**Table 1:** Data collection and measurements.

Collected data and methods		Measurements	Sources
**Patient characteristics**			
	Questionnaire	Year of birth, sex, occupation, level of education, living situation, and date of illness	Patient and patient record
**Staff characteristics**			
	Questionnaire	Year of birth, profession, and time employed in the ward unit	Staff
**Perceived health**			
	Questionnaire	EQ-5D-5L^a^, 1-100 scale [49]	Patient
**Self-efficacy**			
	Questionnaire	SSEQ^b^, 13 items, score 0-3 [47,48]	Patient
**Self-management**			
	Questionnaire	Self-management questionnaire, 8 items, score 1-10 [53]	Patient
**Stroke impact on daily life**			
	Questionnaire	SIS-16^c^, version 2.0, 65 items, 5-point Likert scale [51,52]	Patient
**Participation and experiences of care**			
	Questionnaire	CollaboRATE, 3 items, score 0-9 [50]	Patient
	Semistructured interview	Interview guide: questions about the staff’s daily work in the ward unit, how they are trying to support patients’ self-management, and if the way of treating patients has changed postimplementation	Staff
	Semistructured interview	Interview guide: focusing on how staff invited patients to be involved in their own treatment and how staff gave support for self-management	Patient
	Semistructured interview	Interview guide: questions about how family was involved in the care of the patient and if they saw the staff supporting the patient’s self-management	Family
**Implementation**			
	Questionnaire	NoMAD^d^; three sections covering four dimensions of the normalization process theory; rated on Likert scales [46]	Staff
	Semistructured interview	Interview guide: investigating barriers and facilitators for introducing the intervention in the ward unit and staffs’ views on the implementation process	Staff
	Observation	Observation guide: focusing on the activities taking part, who are involved, and in what context	Staff

^a^EQ-5D-5L: five-level EQ-5D.

^b^SSEQ: Stroke Self-efficacy Questionnaire.

^c^SIS-16: Stroke Impact Scale-16.

^d^NoMAD: Normalization Measure Development questionnaire.

### Data Management

Anonymized data will be entered into SPSS Statistics for Windows (IBM Corp) [54] and securely stored at Dalarna University, Sweden, according to the rules and guidelines for research at the university and the General Data Protection Regulation. All audio files, including recordings of informed consent, will be stored in a secure file at the university. Names, contact information, and identification numbers will be stored separately from the data.

### Analyses

Data will be analyzed between and within the two units, allowing us to understand what factors impact the staff during training and implementation and patients’ health and well-being related to the intervention. This will allow us to understand the implementation process of the intervention within a real-world setting and identify the main factors influencing this process.

Data from the questionnaires will be analyzed using descriptive statistics, and the qualitative data from interviews and observations will be analyzed using thematic analysis [55], supported by NVivo software (QSR International) [56]. The acceptability, adherence, and values of the intervention will be described. The intervention logbook, including field notes and transcribed interviews, will be analyzed using qualitative content analysis. In addition, patients’ experiences of self-management strategies will be analyzed using qualitative content analysis.

### Ethics Approval

Ethical approval was given by the Swedish Ethical Review Authority (2020-02116). Patients and staff have been thoroughly informed of all aspects of the research protocol in which they might be included, and patients have been assigned numbers for anonymization purposes. All data will be collected by phone or using paper questionnaires. Data will be kept in a password-protected database on the research leaders’ institutional server, and paper questionnaires will be kept in a locked container at the university.

## Results

As of March 2022, the first and second phases were performed at site 1. This means that 20 patients have been enrolled and have completed all baseline measurements. A total of 10 significant others have been interviewed. Observations of the organization and staff interviews have been conducted. The intervention has been performed online by the Bridges team. A total of eight workshops have been conducted online. The researchers have observed workshops, during which they made field notes. Data are being transferred to SPSS and analysis will start when the implementation is completed. However, phase 3 has not started yet, since the implementation had not been completed.

The second site has been recruited, and data collection of baseline data will begin in spring 2022 and will continue for another 6 to 12 months. The intervention is planned to take place in early autumn 2022.

## Discussion

### Principal Findings

As one of the first studies to examine the feasibility and implementation of a stroke self-management program integrated into a Swedish stroke setting, this study will contribute to (1) a better understanding of the benefits of a self-management approach that specifically targets changing the ways that care providers work with and support patients with stroke and (2) exploring the value of embedding implementation science approaches into the study of complex interventions in stroke, in order to better inform policy makers’ decisions about, and implementation of, stroke service delivery.

The qualitative data from the interviews will provide valuable insights as to whether, from a patient perspective, self-management support provided through an adapted Bridges program will reflect greater perceptions of more person-centered care. The study will also provide data to explore whether there have been improvements in perceived health, as well as corresponding improvements according to the underpinning principles and fidelity of Bridges, following a period of staff working in the “Bridges way.”

From a clinical perspective, the study will provide new knowledge of whether using a self-management support program based on the concept of self-efficacy and principles to support self-efficacy for self-management will improve rehabilitation through a new integrated way of working into existing health care interactions where people’s needs and skills are taken into account during the first interaction. The intervention is integrated into the way of working rather than being perceived as an add-on to care. We hope that the intervention will foster a better anchoring of patients’ and families’ needs in the rehabilitation process, reducing the risk that the health care professional is the one who defines the problems. In addition, the intervention is intended to favor more active participation by the patient and family and all members of the rehabilitation team; this will be explored through the qualitative and quantitative data from the study.

Having used implementation science approaches throughout the study design, we expect to obtain deep insights into the implementation process from an individual to a macro perspective. Although each of the domains of the NPT may be necessary for ultimate success, health professional associations have a distinct role, from a macro perspective, in both coherence and cognitive participation, essentially in the sensemaking and relational work required to enact change. Considering the NPT at this level may strengthen the theory and suggest unique factors when looking beyond individuals within a team. Findings from this study will inform best practices guidelines by providing empirical data on effective implementation processes of self-management support, both in general and with relevance for scale-up and spread of this approach to other services in the Swedish context.

The dissemination of results will not only take place through academic publications, but will also focus on communicating research results to practitioners and the public in appropriate trade journals, newspapers, and meeting forums, both in face-to-face meetings and online. Dissemination of popular science articles outside the academic world will take place by various members of the research group.

We anticipate that the findings of the study, overall, will address the current call for health care to move toward more self-management and generate new knowledge about what contributes to successful and sustainable self-management support for people with long-term conditions, which is still underresearched in Swedish health care. The project has advantages, as it makes use of existing service time and can be used by all health care professionals in the context of the patient. From a patient perspective, self-management support will mean more person-centered care focused on the patient’s ability and opportunity to take an active part in the care of their health in everyday life. The patient will have support beyond that of earlier self-management interventions that relied too heavily on an individualistic approach, and that focused on personal agency without considering personal circumstances, including social capital, networks, health literacy, ethnicity, and cultural aspects [26].

### Strengths and Limitations

The proposed study design is a case study design, and the impact on patients’ health, well-being, and self-efficacy will need to be tested with a more experimental design. However, our case study research will contribute to a comprehensive and multifaceted exploration of an intervention in a natural uncontrolled setting. We will collect quantitative and qualitative data to understand the implementation process, context-dependent insights, and the feasibility of the intervention when adapted to the Swedish context.

### Conclusions

This study represents a unique, highly relevant, and innovative opportunity to maximize knowledge and minimize practice gaps in rehabilitation stroke care. The study will produce robust data on the effectiveness of the intervention and in-depth data on the contextual factors and mechanisms related to its effectiveness, for whom it is effective, and how it is effective. Participating health care providers will gain the resources to engage patients and families and develop their interprofessional self-management skills, which are crucial to meeting patients’ needs and to significantly improving patient self-management support and the rehabilitation process.

## Appendix

Multimedia Appendix 1 Description of the study phases.

Multimedia Appendix 2 Logic model of the SELMA (Self-Management) project.

Multimedia Appendix 3 Peer Review Report from Forte: Forskningsrådet för hälsa, arbetsliv och välfärd, Sweden (Forte: Swedish Research Council for Health, Working Life and Welfare).

## Abbreviations

Bridges: Bridges Self-Management
EQ-5D-5L: five-level EQ-5D
MRC: Medical Research Council
NoMAD: Normalization Measure Development questionnaire
NPT: normalization process theory
RCT: randomized controlled trial
SELMA: Self-Management
SIS-16: Stroke Impact Scale-16
SPIRIT: Standard Protocol Items: Recommendations for Interventional Trials
SSEQ: Stroke Self-efficacy Questionnaire
