# Supplementary figures and images for: Tailoring and Evaluating an Intervention to Support Self-management After Stroke: Protocol for a Multi-case, Mixed Methods Comparison Study
Source: JMIR Res Protoc. 2022 May 6;11(5):e37672. doi: 10.2196/37672 (PMC9123550; doi:10.2196/37672)

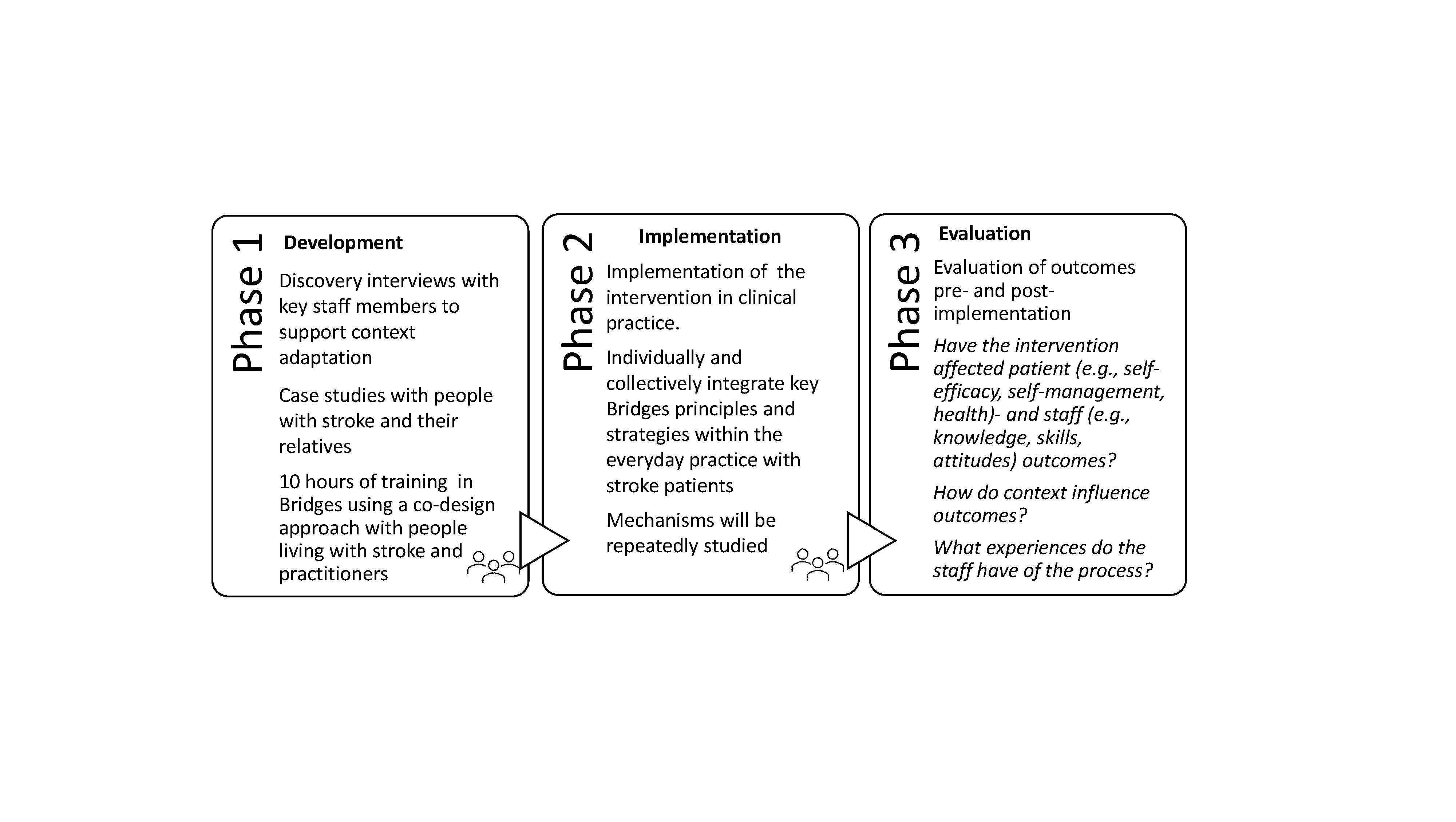

Supplement: Multimedia Appendix 1 [file resprot_v11i5e37672_app1.png]

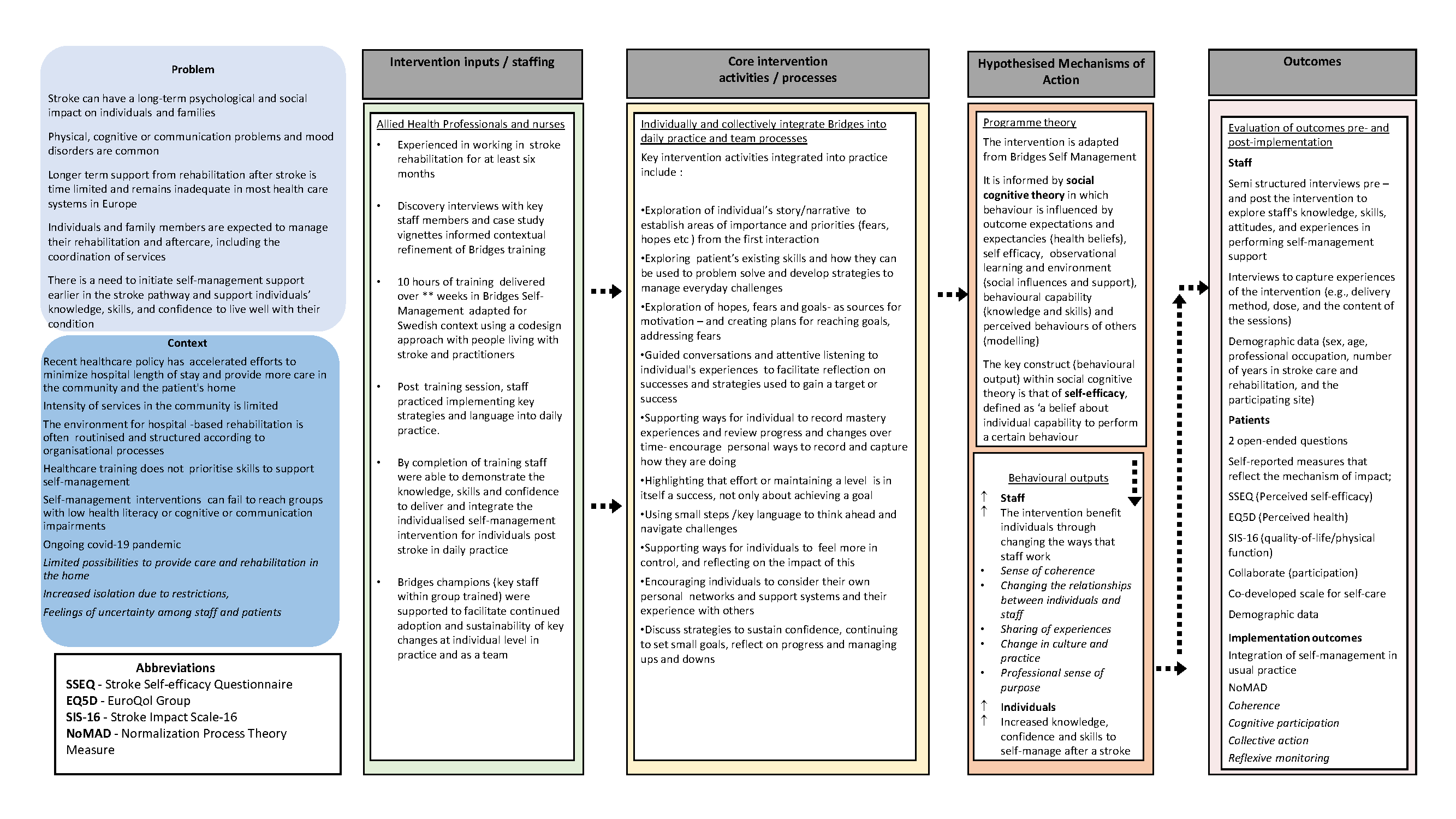

Supplement: Multimedia Appendix 2 [file resprot_v11i5e37672_app2.png]
