# Supplementary material for: Tailoring and Evaluating an Intervention to Support Self-management After Stroke: Protocol for a Multi-case, Mixed Methods Comparison Study
Source: JMIR Res Protoc. 2022 May 6;11(5):e37672. doi: 10.2196/37672 (PMC9123550; doi:10.2196/37672)
Supplement: Multimedia Appendix 3 [file resprot_v11i5e37672_app3.pdf]

FORTE:
Forskningsrådet för hälsa, arbetsliv och välfärd

Beredningsgruppens yttrande

2020-01236	Marie Elf	Beredningsgrupp: ImpForsk20

Utlysningsnamn: Implementeringsforskning 2020

Bidragsform: Projekt

Projekttitel (svenska): Implementation of self-management - the SELMA project

Sökt inriktning: Implementeringsforskning

Bedömning

Syfte, frågeställningar, teorianknytning, bakgrund och projektets originalitet

This very focused care-improvement project in two dedicated stroke rehabilitation units, aims to train the profession unit staff in the use of SELMA, a complex care intervention designed to make health care staff better able to coach their patients in self-management, goal-setting and attainment, and autonomy. The intervention will be implemented over some months after an initial staff training period, and then applied to 40 patients over the next several months, after a "control" pre-intervention period. Identical multiple outcome measures, almost all of them from self-report questionnaires -- self-efficacy, well-being, health related quality of life, etc. -- will be made on both pre-and post- intervention patients (40 of each, per unit). Most of the analysis will be of qualitative process data, together with simple inferential statistics performed on the pre-/post- test comparison groups in each unit. The intervention is deeply rooted in both psychological theory and organisational-change theory, and the project is quite original, and it is clear that the applicants have worked on care-improvement in stroke rehabilitation with this or similar interventions in the past.

Studiedesign, metoder för datainsamling och analys

There are two methodological weaknesses of this proposal. The first is the low-quality study design to be used to evaluate all
the effects (good and bad) of the intervention: a simple before/after comparison in only two relatively closed sets of health professionals in two specialist stroke rehab units, and a limited number (80) of their patients. The applicants will need to guard against the following threats to internal validity that often undermine this study design: a) Hawthorne/"Attention" Bias (especially for the many process measures to be collected from the healthcare practitioners, none of whom will presumably be
blinded to the before-after status of their responses that signifies intervention allocation, and most of whom will be heavily invested, by their SELMA training programme, in its success; b) temporal /secular trend confounding -- e.g. what if some other unforeseen factor (such as a coronavirus outbreak!) comes along between the pre- and post- measurements? ; c) inability to control for other factors which may influence the outcomes measured, and could well be differential during the before/after periods, many of them likely acting at the stroke-unit or practitioner level. Secondly, there is no sample size calculation, with the 40 patients set as the recruitment target in both pre- and post-phases not justified at all, and quite unlikely to be adequate for some less common outcomes to be measured. Nonetheless, the panel felt that the strong track-record and relevant experience of the research team would be able to these relative methodological shortcomings in the design.

Genus- och mångfaldsperspektiv i forskningens innehåll

This is a strength of the proposal, in that the SELMA intervention package clearly works to optimize individual patient progress towards autonomy and self-efficacy, independent of gender/ race/ethnicity, etc.

Genomförbarhet

The study is feasible to carry out, but it has a significant risk of not being easily replicable, because the intervention to be tested is indeed complex -- and rather heavily "psychologized" for many health-care professionals to find it easy to comply with, especially in a busy stroke unit, typically filled with many cognitively-, linguistically- and motor-impaired patients who demand a lot of attention and understanding. This means that a stroke unit able and willing to go through such an onerous QI process, largely to achieve psychometrically measured outcomes, is unlikely to be a typical stroke unit. The applicants will need to pay special attention to how their intervention can be ramped up for broader application, if it is successful in this study setting.

Relevans, samverkan och nyttiggörande

This an implementation of a very complex and demanding training package for healthcare staff, to improve patient self-care, self-efficacy and autonomy in two stroke rehab units. While the project will contribute meaningfully to the implementation science literature, it will need careful attention to threats to both its internal and external validity if its potential is to be maximized.
Regarding public and patient/family engagement, more effort could have been made by the applicants to plan for the creation of a strong and diverse stakeholder advisory committee to the project.

Sammanfattande bedömning

Despite some weaknesses identified by the panel, the panelists were willing to give the very experienced investigator team the benefit of the doubt, given this proposal's strong theoretical base, and the researchers excellent track record and deep familiarity with the study setting.

Förslag till beslut (bevilja, reserv, avslå)

Approve
